# Case report: subacute tetraplegia in an immunocompromised patient

**DOI:** 10.1186/s12883-017-0814-5

**Published:** 2017-02-10

**Authors:** Daniel Zeller, Anke Heidemeier, Götz Ulrich Grigoleit, Wolfgang Müllges

**Affiliations:** 1 Department of Neurology, University of Würzburg, Josef-Schneider-Str. 11, 97080 Würzburg, Germany; 2 Department of Radiology, University of Würzburg, Josef-Schneider-Str. 11, 97080 Würzburg, Germany; 3 Department of Internal Medicine II, University of Würzburg, 97080 Würzburg, Germany

**Keywords:** Tetraparesis, Motor cortex, CMV encephalitis, Case report

## Abstract

**Background:**

Clinical reasoning in Neurology is based on general associations which help to deduce the site of the lesion. However, even “golden principles” may occasionally be deceptive. Here, we describe the case of subacute flaccid tetraparesis due to motor cortical lesions. To our knowledge, this is the first report to include an impressive illustration of nearly symmetric motor cortical involvement of encephalitis on brain MRI.

**Case presentation:**

A 51 year old immunocompromized man developed a high-grade pure motor flaccid tetraparesis over few days. Based on clinical presentation, critical illness polyneuromyopathy was suspected. However, brain MRI revealed symmetrical hyperintensities strictly limited to the subcortical precentral gyrus. An encephalitis, possibly due to CMV infection, turned out to be the most likely cause.

**Conclusion:**

While recognition of basic clinical patterns is indispensable in neurological reasoning, awareness of central conditions mimicking peripheral nervous disease may be crucial to detect unsuspected, potentially treatable conditions.

## Background

Connecting clinical findings to neuroanatomy is a basic principle of neurologists’ thinking. One of the “golden principles” is the simple association flaccid paresis plus muscle atrophy pointing to peripheral nerve disease. However, even basic principles must be questioned occasionally. A rather common example is that of patients waking up with a drop hand – although clinically suggestive of a peripheral origin (i.e. radial nerve palsy), the alternative cause may be an acute central nervous system lesion (i.e. “hand knob” infarction) [1, 2]. The other way around, fulminant courses of inflammatory demyelinating polyneuropathy (Guillain-Barré syndrome, GBS) may mimic brain death [3]. Here, we describe the instructive case of a patient who subacutely developed flaccid tetraparesis due to bilateral motor cortical lesions, illustrated by striking findings on brain magnetic resonance imaging (MRI).

## Case presentation

Six weeks after stem cell transplant for myelodysplastic syndrome, a 51 year old caucasian patient developed a high-grade pure motor flaccid tetraparesis within a few days. While cognitive and brainstem functions – including the function of facial muscles – were preserved, the patient was tetraplegic except for minimal finger movements of the left hand. The deep tendon reflexes were absent, and Babinski testing revealed mute soles on both sides. There was considerable generalized muscle atrophy which had already been noticed at first neurological assessment five days before transplant. At that occasion, however, the patient had presented without any paresis.

Development of tetraparesis within several days along with absent deep tendon reflexes might be indicative of GBS. However, the simultaneous and generalized rather than ascending development of tetraparesis is uncommon in GBS. In addition, ongoing strong immunosuppression by cyclosporine A and mycophenolate mofetil made the new occurrence of an autoimmune disorder unlikely. Therefore, taking into account that the patient had been treated in ICU for pneumonia with respiratory failure before, critical illness polyneuromyopathy was suspected.

**Fig. 1:**
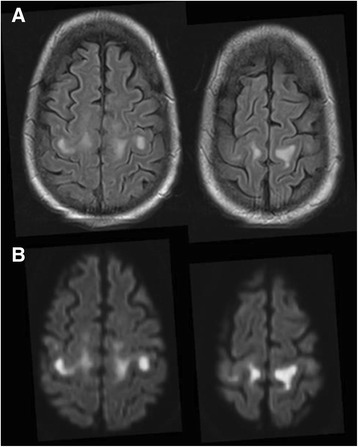
Magnetic resonance imaging in a patient with subacute flaccid tetraplegia. FLAIR images show a hyperintense signal confined to subcortical precentral gyrus on both hemispheres **a**. This hyperintensity is associated with high signal on diffusion-weighted imaging **b**, without ADC correlate (not shown)

A few days later, however, an acute confusional state prompted brain MRI. Fluid-attenuated inversion recovery (FLAIR) imaging revealed symmetrical hyperintensities strictly limited to the subcortical precentral gyrus (Fig. 1a). Correspondingly, diffusion-weighted imaging (DWI) showed signal hyperintensity (Fig. 1b), along with normal ADC and without any contrast enhancement. Six days before transplant, the patient had received cranial MRI without any pathological finding. Thus, tetraplegia was attributable to new symmetrical CNS lesions.

Stroke, for example due to cardiac embolism, was not supported by normal ADC. “Stroke mimics” with positive DWI, but without lesion hypointensity on the ADC map, include active MS plaques, cerebral abscess, and viral encephalitis [4]. The spatial pattern of the MRI lesions appeared virtually incompatible with MS, and the absence of a mass effect or rim enhancement rendered bacterial cerebritis or cerebral abscesses very unlikely [5]. Taken together, subacute development of tetraparesis along with new subcortical lesions in an immunocompromised patient were highly suggestive of infection by or reactivation of neurotropic viruses. Sole motor affection may make poliovirus highly suspicious [6], particularly in the light of recent reports of polio cases in Germany [7]. However, given the patient’s vaccination against polio virus, this differential diagnosis was considered rather unlikely.

Spinal tap revealed a normal cell count (1 leucocyte/mm^3^, normal range 0–4) along with slightly elevated protein (56.2 mg/dl, normal ≤50) and albumine (50.9 mg/dl, normal ≤35). CSF PCR was negative for herpes simplex virus type 1/2, varizella zoster virus, Epstein-Barr virus, cytomegalovirus, human herpes virus six, JC virus, adenovirus, enterovirus, poliovirus, and bacterial DNA (universal primer). However, repeat serum PCR for CMV showed a progressively increasing number of up to 8,600 copies/ml, starting shortly prior to the development of tetraparesis.

Sensitivity of CSF PCR for CMV encephalitis is generally believed to be high, yet unequal to 100% as far as known from comparison with viral isolation from autopsy samples [8]. Moreover, CSF virus PCR might initially be false-negative, with increasing probability of a positive PCR when there is a time frame of 3 – 14 days between symptom onset and lumbar puncture [9]. Therefore, while the laboratory findings were unable to prove CMV encephalitis, they were compatible with this suspected diagnosis. Accordingly, intravenous ganciclovir treatment (5 mg per kg per day over two weeks) was initiated. Over the following three weeks, serum PCR for CMV turned negative. Tetraparesis slowly improved, with still moderate pareses of the upper (between MRC 3 and 4), but severe pareses of the lower (between MRC 1 and 2) extremities at neurological reassessment four weeks after symptom onset. Unfortunately, in the further course the patient developed a septic syndrome, most likely owing to a pulmonary focus, and exspired due to septic multiple organ failure. Autopsy was prohibited.

## Discussion and Conclusions

Though primarily suggestive for peripheral nervous disease, subacute flaccid tetraplegia may reflect pure motor cortical paresis [10, 11]. In our case, MRI pointed to an encephalitis limited to the bilateral subcortical precentral gyri. Thus, while recognition of basic patterns of clinical findings is indispensable in clinical reasoning, awareness of mimics may help to detect unsuspected treatable conditions.

## Abbreviations

ADC: Apparent diffusion coefficient
CMV: Cytomegalovirus
CNS: Central nervous system
CSF: Cerebrospinal fluid
DNA: Deoxyribonucleic acid
DWI: Diffusion-weighted imaging
FLAIR: Fluid-attenuated inversion recovery
GBS: Guillain-Barré syndrome
MRI: Magnetic resonance imaging
MS: Multiple sclerosis
PCR: Polymerase chain reaction
